# Uncovering convergence and divergence between autism and schizophrenia using genomic tools and patients’ neurons

**DOI:** 10.1038/s41380-024-02740-0

**Published:** 2024-09-05

**Authors:** Eva Romanovsky, Ashwani Choudhary, David Peles, Ahmad Abu-Akel, Shani Stern

**Affiliations:** 1 Sagol Department of Neurobiology, Faculty of Natural Sciences, University of Haifa, Haifa, Israel; 2 Institute of Pathology, Heidelberg University Hospital, Heidelberg, Germany; 3 School of Psychological Sciences, University of Haifa, Haifa, Israel; 4 The Haifa Brain and Behavior Hub, University of Haifa, Haifa, Israel

**Keywords:** Neuroscience, Autism spectrum disorders, Schizophrenia

## Abstract

Autism spectrum disorders (ASDs) are highly heritable and result in abnormal repetitive behaviors and impairment in communication and cognitive skills. Previous studies have focused on the genetic correlation between ASDs and other neuropsychiatric disorders, but an in-depth understanding of the correlation to other disorders is required. We conducted an extensive meta-analysis of common variants identified in ASDs by genome-wide association studies (GWAS) and compared it to the consensus genes and single nucleotide polymorphisms (SNPs) of Schizophrenia (SCZ). We found approximately 75% of the GWAS genes that are associated with ASD are also associated with SCZ. We further investigated the cellular phenotypes of neurons derived from induced pluripotent stem cell (iPSC) models in ASD and SCZ. Our findings revealed that ASD and SCZ neurons initially follow divergent developmental trajectories compared to control neurons. However, despite these early diametrical differences, both ASD and SCZ neurons ultimately display similar deficits in synaptic activity as they mature. This significant genetic overlap between ASD and SCZ, coupled with the convergence towards similar synaptic deficits, highlights the intricate interplay of genetic and developmental factors in shaping the shared underlying mechanisms of these complex neurodevelopmental and neuropsychiatric disorders.

## Introduction

Autism spectrum disorders (ASDs) are a complex and heterogeneous group of neurodevelopmental disorders that affect approximately 1% of children globally and vary in degree of severity [1]. According to the American Psychiatric Association’s DSM-5 (Diagnostic and Statistical Manual of Mental Disorders, Fifth Edition) criteria, abnormal social interaction, repetitive behavior, and delayed language and cognitive skills are the main characteristic symptoms used to diagnose ASD at an early age [2]. ASD is one of the highly heritable neuropsychiatric disorders (83%) [3], and large-scale genomic studies e.g., SPARK [4] (Simons Foundation Powering Autism Research) have led to the implications of hundreds of genes that may play important roles in the manifestation of ASD [1, 5]. Schizophrenia (SCZ), another complex neuropsychiatric disorder, is associated with the presence of core symptoms that have been classified along negative (e.g., blunted affect, asociality) and positive (e.g., delusions and hallucinations) dimensions, as well as cognitive disorganization, and its prevalence has been estimated at 0.35% worldwide [6]. SCZ similarly to ASD has high heritability estimates (~80%) with a clear difference in the age of onset (manifestation of SCZ is generally in adulthood) [7], and it too has been associated with a large number of genetic variants (>170) [8, 9].

The nature of the relationship between ASD and SCZ has been the subject of intense debate [10]. While diagnostically independent, clinical reports indicate that ASD co-occurs with SCZ and vice-versa at rates higher than in the general population [11], as well as with other developmental and neurological disorders including, among others, intellectual disability, attention-deficit hyperactivity disorder (ADHD), anxiety, depression, and epilepsy [12]. Moreover, multidimensional evidence from phenotypic, behavioral, neuroimaging, environmental, and genetic substrates point to considerable overlap between ASD and SCZ [11, 13]. For example, a meta-analysis of GWAS data of eight psychiatric disorders involving subjects of European ancestry found significant genetic correlations between the disorders, including between ASD and SCZ [14]. Clinical symptoms based on DSM-5 also appear to overlap, especially with respect to impairments in social interaction in ASD and the negative group of symptoms such as social withdrawal and reduced communication in SCZ [11]. Finally, meta-analyses of neuroimaging studies have also suggested alteration of common brain regions like anterior cingulate volume, anterior insular cortex, cerebellum [15–17], and functional networks (e.g., sensorimotor, cognitive control) among neuropsychiatric disorders, including SCZ and ASD [17–19].

Given the considerable overlap observed between SCZ and ASD, identifying common pathways can offer valuable insights that could potentially inform more effective and targeted interventions for both disorders. Rare structural variants (or copy number variations) such as deletion at loci 22q11.2 and 15q13.3 or duplication at 16p11.2 are associated with both SCZ and ASD and also with other developmental disorders [18, 20]. However, the majority of these rare variants stretch across large genomic regions housing multiple genes and hence the position of specific mutations at the genic or allelic level is difficult to infer. Most of the prior studies have focused on the shared rare variants and their pleiotropic effects in the development of SCZ and ASD [14, 19, 21, 22]. However, for a better understanding of SCZ and ASD, further characterization of the overlapping genetic and pathophysiological processes is required, particularly for the GWAS-discovered common variants [23].

Over the last decade, induced pluripotent stem cells (iPSC)-based models of SCZ and ASD [24–32] have provided important clues about the pathophysiological processes affected in neural cells, especially since the animal models do not fully recapitulate the complex genetics and clinical features of neuropsychiatric disorders [33]. We recently performed a quantitative meta-analytical study of the progress done in the field of SCZ genetics and the findings of SCZ phenotypes in iPSC-derived neural cells [7]. Here, in this study, we found that 75% of the GWAS genes are common between ASD and SCZ by analyzing datasets from diverse ancestries. Examining this overlap in greater depth, we also identified overlapping and unique SNPs between ASD and SCZ focusing on strongly associated GWAS genes. To understand the functional role of these common genetic variants in ASD, we compared the GWAS genes with those in the Developmental Brain Disorder Database (DBD) and identified 30 common genes that are highly expressed in the cerebellum, cerebral cortex, basal ganglia, and hippocampus compared to other regions. Additionally, we analyzed around 50 iPSC-based ASD studies and compared the results with previously published SCZ iPSC studies [7] to gain insights into the pathophysiology and progression of both disorders. We discovered that ASD and SCZ iPSC patient-derived neurons exhibit distinct and somewhat opposite neurophysiological properties at early stages of the differentiation but develop similar synaptic deficits at more mature stages. Thus, using a quantitative meta-analytical approach to both genomics and iPSC-based disease modeling, we have elucidated the similar and distinct features between ASD and SCZ.

## Methods

### Ethics approval and consent to participate

All methods were performed following the relevant guidelines and regulations. The GWAS datasets analyzed were collected from the *GWAS catalog* which is a public database maintained by NHGRI-EBI [34]. The iPSC-based ASD modeling data were collected from previously published studies (Supplementary Table 1) following the relevant guidelines and approved by ethics committees.

### Genetic variant analysis and standardization of gene nomenclature of autism spectrum disorder (ASD), schizophrenia (SCZ), and bipolar disorder (BD) genes

The genetic variant analysis and the standardization of gene nomenclature were performed according to Choudhary et al. [7]. Trait statistics were retrieved from the NHGRI-EBI Genome-Wide Association Study (GWAS) Catalog (last accessed May 2024) [35] for the study of ASD (EFO_0003756), SCZ (EFO_0004609), and BD (EFO_0009963). A total of 17 publications with 305 genes related to ASD; 86 publications with 1119 genes related to SCZ; and 77 publications with 694 genes related to BD were reported. The *SNP_GENE_IDS* column was utilized to identify the Ensembl IDs of genes that contain ASD-related reference SNPs (rs, SNP-risk allele) within their sequence. When several Ensembl IDs were attributed to one gene in a single publication, they were unified and the gene symbol was counted once in each publication to reduce noise from the data because Ensembl IDs of the same gene symbol had overlapping loci in most cases. Furthermore, care was taken to ensure that each publication was counted only once since some publications had multiple entries and thus the same Ensembl IDs. For each gene variant, the frequency was calculated by counting how often it was reported in a publication with a *p*-value of <0.01. The strongly associated genes for ASD are those counted in more than three publications (*n* = 23) and for SCZ those counted in more than five publications (*n* = 105).

The full dataset from the Developmental Brain Disorder Gene Database (DBD) [36] (we used a previous version where six neurological disorders were included) was downloaded to extract a list of genes associated with ASD. According to DBD, 672 genes have been linked to ASD. The Ensembl Data (Biomart, 2022-08-25) [37] was accessed to acquire a list of Ensembl IDs and their corresponding gene name. The chosen dataset was *Human Genes (GRCh38.p13)* from *Ensembl Genes 107 *and the attributes selected were *Gene Stable ID* and *Gene Name*. Additionally, the database of Human Gene Nomenclature Committee (HGNC) gene symbols from EMBL-EBI Public FTP [http://ftp.ebi.ac.uk/pub/databases/genenames/hgnc/tsv/hgnc_complete_set.txt] (2022-08-25) was downloaded which contained approved gene symbols, previous gene symbols, alias gene symbols and the related Ensembl ID for each gene. The gene nomenclature was standardized for GWAS and DBD gene symbols using HGNC as described in Choudhary et al. [7]. The method involves searching for the Ensembl ID in both the Biomart results and the HGNC database. If the Ensembl ID is found in the HGNC database, the matching approved symbol from the HGNC database is used. If the Ensembl ID is not found in the HGNC database, the Biomart symbol is compared to the list of approved HGNC symbols. If the symbol is an approved HGNC symbol, it is used. If the symbol is not an approved symbol, it is searched in the list of previous gene symbols in the HGNC database. If the symbol matches a previous symbol of a gene, the currently approved HGNC symbol is used. This method facilitates the matching of HGNC-approved symbols to all Ensembl identifiers with no potential for confusion. For ASD genes of GWAS, the identifier *LINC02163* was replaced by the approved symbol *NIHCOLE*. Similarly, for ASD genes of DBD, the identifier *C12ORF57* was not located in either the HGNC nor the Biomart symbols, and the identifier *KIAA0100* was substituted by the approved symbol *BLTP2*. The gene symbols of SCZ were already standardized by Choudhary et al. [7].

### Data analyses

All statistical analyses were performed by using R (version 4.1.2 (2021-11-01)) [38] and MATLAB (version 9.12.0.2009381 (R2022a) Update 4, 2022-07-7) [39].

### Investigating the commonalities between ASD and SCZ genes; single nucleotide variants (SNPs)

To find common genes, the intersection of ASD genes from GWAS and DBD was formed first (*n* = 30 genes), followed by the intersection of ASD and SCZ genes from GWAS (*n* = 78 genes). Overlapping genes were represented as word clouds using MATLAB. Genes were color-coded so that their overlap with the corresponding gene list could be seen. The frequency of genes associated with ASD that appeared more than three times in publications was visualized using MATLAB. Subsequently, the commonalities between ASD and SCZ were further investigated. For this purpose, the most strongly associated genes in ASD (≥3 publications) and SCZ (≥5 publications) were overlapped to create a common gene list (*n* = 17). The SNPs of the common genes as reference SNP (rs) were taken from the GWAS catalogs of ASD and SCZ (*SNPS* column). The common genes were matched with the SNPs and merged into one heatmap. ASD (red) and SCZ (green) were marked when the SNP was detected. The *oncoPrint()* function from the R package *ComplexHeatmap* [40] was used to generate the heatmap. In addition, all chromosomes were represented as a circular layout to mark the genomic position of detected SNPs. The human reference genome assembly GRCh38 was used (Supplementary Fig. 3). The *RCircos* R package [41] was employed for this purpose. The maturation and neuron development scheme in iPSC models of ASD and SCZ has been simplified and created with Biorender.com. The scheme was referred to previous iPSC studies [42–44], including our previously reported studies [25, 45–47].

### Analysis of RNA expression levels of ASD genes across brain regions

RNA consensus tissue gene data (*rna_tissue_consensus.tsv*) was downloaded from The Human Protein Atlas (HPA, proteinatlas.org) [48]. This data set provided a summary of normalized expression levels for genes across 54 different tissues, based on transcriptomics data from HPA and Genotype-Tissue Expression (GTEx). The data included an Ensembl gene identifier (*Gene*), analyzed sample (*Tissue*) and normalized expression value (*nTPM*) for each gene. It was based on the HPA version 22.0 and Ensembl version 103.38.

First, the data set was subset by the overlapping ASD genes of GWAS and DBD. The average gene expression was calculated for each tissue, filtered by brain regions including the amygdala, basal ganglia, cerebellum, cerebral cortex, hippocampal formation (hippocampus), hypothalamus, medulla oblongata (medulla), midbrain, pons, spinal cord, and thalamus. Biorender.com was utilized to assign color coding based on the ranking of average expression level, with red signifying a high expression level and violet representing a low expression level. The illustration is not a precise representation of the anatomical structure of the brain but rather a schematic one. The colored areas were used to demonstrate the different brain regions.

### The genetic pathways of ASD explored using gene set enrichment analysis

A gene set enrichment analysis was performed on the most commonly observed ASD genes and those ASD genes that overlapped in both GWAS and DBD to ascertain the gene networks or biological pathways implicated in ASD. The CytoScape STRING app [49] was used to create a gene set network and to perform the functional enrichment analysis. The terms of 15 categories, including Gene Ontology Cellular Component (GO) and Monarch Phenotypes (EFO, HP), were analyzed. The results of the analysis were considered significant when the false discovery rate (FDR) adjusted *p*-value was less than 0.05 using the Benjamini-Hochberg procedure. Significant terms associated with ASD or SCZ were highlighted in the gene set network.

### Disease phenotypes in ASD-induced pluripotent stem cells (iPSC) and a comparison to SCZ iPSC models through data visualization

A total of 51 ASD-iPSC-related publications were evaluated (Supplementary Table 1). Listed studies by Chiffre et al. [50] were also included. The overview of numerous collected data was divided into several pie charts. Subsequently, the individual phenotypes and their morphological and functional changes during the differentiation of ASD-iPSCs were compiled as a heatmap. The regulation or effect strength has been color-coded. The green color represents low regulation (↓) and the red color represents high regulation (↑) of reported disease phenotypes. The color bar represents the number of ASD-iPSC-based models in which the corresponding regulation was observed. The R programming language was used to create the pie charts and heatmap, and the R packages *ggplot2* [51] and *ggpubr* [52] were used for this purpose.

## Results

### Linking the genome-wide associated genes of ASD and SCZ

**Fig. 1:**
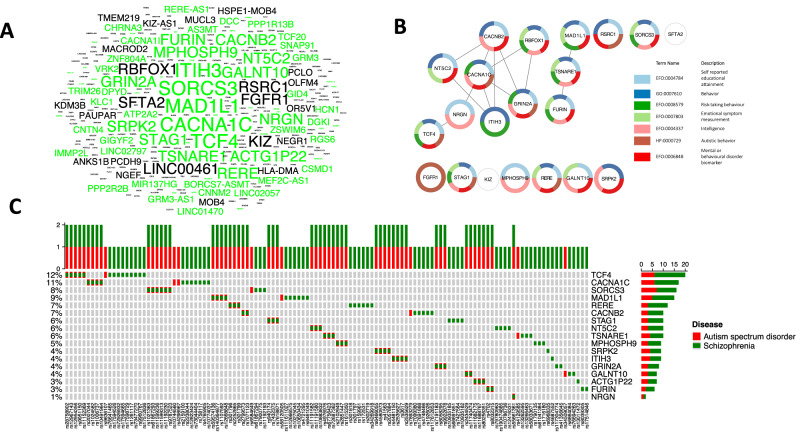
ASD and SCZ GWAS genes. **A** The word cloud of GWAS-reported genes associated with ASD. GWAS-reported genes associated with SCZ are highlighted in green. The font size indicates the frequency of gene mentions in publications, with a larger font size indicating a higher number of reported occurrences. The strongly associated genes with ASD have been plotted in Supplementary Fig. 2A. **B** Gene network of the strongly associated GWAS genes with ASD. Functional analysis revealed significant pathways indicating Autistic behavior. Connected genes imply interactions. **C** Color map for shared SNPs between ASD and SCZ in GWAS. The intersection of the strongly associated genes (*n* = 17) in ASD and SCZ and their 122 SNPs has been visualized.

Using the GWAS Catalog [35], we searched the database for “Autism Spectrum Disorders” and identified the highly associated genes when collecting the data from 17 studies. We included the studies from diverse ethnic groups/ancestries as plotted in Supplementary Fig. 1A. Approximately, 35% reported European ancestry followed by African (~12%) and East Asian ancestry (~9%). Moreover, approximately 27% of the studies were lacking ancestry information. We then analyzed the genetic variants associated with ASD that appeared in publications with a *p*-value of <0.01 (Fig. 1A). Additionally, and as analyzed in our previous study [7], we checked which of the genes that showed associations with ASD are also associated with SCZ using the GWAS Catalog as a reference database. Out of the 305 reported ASD GWAS genes, 239 (78%) were also found to be associated with SCZ (see methods). We further selected 105 SCZ GWAS genes from the total 1119 SCZ genes in the GWAS database (also see methods) based on their appearance in 5 or more publications. Of these 105 SCZ genes, 78 (74%) were also associated with ASD (Fig. 1A).

Next, we established criteria for strongly associated genes, defining strongly associated as being reported in more than 3 publications for ASD and more than 5 publications for SCZ (see methods). Notably, 74% (17/23) of the strongly associated genes in ASD were also associated with SCZ (Supplementary Fig. 2A). Some of these strongly associated genes are crucial to neuronal function and integrity. These include genes encoding for the subunits of voltage-gated calcium channels (CACNA1C, CACNB2, CACNA1l, and CACNA1I) [53]; TSNARE1, a part of SNARE complex and important for synaptic vesicle release and neurotransmitter function of neurons [54]; GRIN2A, a subunit of the NMDA receptor especially critical for neurotransmission in excitatory neurons [55]; SORCS3, a specific receptor of nerve growth factor (NGF) especially in hippocampus and cortex [56]. Some genes with essential cellular and molecular functions were also found to be highly associated e.g., MAD1L1, a component of spindle assembly checkpoint crucial for the segregation of chromosomes during mitosis [57]; SRPK2, a serine /arginine-rich protein kinase and important in the regulation of mRNA splicing [58]; ITIH3, a member of the inter-alpha-trypsin inhibitor family and involved in the stabilization of the extracellular matrix [59]. Previous studies have also reported a high genetic correlation between SCZ and BD [60, 61]. We also analyzed the overlap of genes associated with ASD, SCZ, and BD identifying 64 genes common to all three disorders including CACNA1C, MAD1L1, and SORCS3 (Supplementary Fig. 1B; also see methods).

We then explored the functional connections among the 23 genes most prevalent in ASD GWAS, creating a visual gene network using STRING functional enrichment analysis (see Fig. 1B). Sixteen of these genes showed significant enrichment for “Self-reported educational attainment” (*p* = 4.3e-17), and 15 genes showed similar enrichment for “Behavior” (*p* = 3.9e-16). Notably, CACNA1C was directly connected to five other genes: CACNB2, GRIN2A, NRGN, RBFOX1, and ITIH3. Most of these genes are known to be associated with calcium-mediated activity of neural cells [62]. Additionally, TCF4 and NT5C2 were interconnected in this network. Common significant terms for these genes included “Intelligence,” “Mental or behavioral disorder biomarker,” and “Risk-taking behavior.” Another distinct network comprised the genes MAD1L1, TSNARE1, and FURIN, which are involved in secretory pathways, including synaptic vesicle release and fusion. These genes were notably associated with “Risk-taking behavior,” “Educational attainment,” and “Mental or behavioral disorder biomarker.”

### SNPs that are common or unique in SCZ and ASD

Subsequently, we aimed to investigate whether specific SNPs are frequently associated with either ASD or SCZ within the 17 genes common to both disorders (Fig. 1C). For example, for the TCF4 gene (a transcription factor), nine SNPs are associated with SCZ only, one with ASD only, and five SNPs are common. Similarly, for the CACNA1C gene, seven SNPs are associated with SCZ only, two with ASD only, and four with both ASD and SCZ. Overall, 48% of SNPs are associated with SCZ only, 45% are common SNPs, and only 7% of the SNPs are associated exclusively with ASD, possibly due to the comparatively fewer ASD GWAS conducted compared to SCZ. Further studies in larger cohorts may determine whether these SNPs are more specific for ASD or SCZ.

We also mapped the regions in the different chromosomes that are associated with ASD-specific genetic variants (Supplementary Fig. 3A) and both with ASD and SCZ (Supplementary Fig. 3B). Chromosome 3 (10 SNPs), 7(9 SNPs), and 10 (9 SNPs) had the highest number of specific SNPs reported for ASD. Chromosome 7 and 10 (9 SNPs each) had the highest number of common SNPs shared in ASD and SCZ. Overall, 63 SNPs have been reported from 17 strongly associated genes common between ASD and SCZ while there were 82 SNPs from 23 genes strongly associated with ASD (Supplementary Fig. 3A, B).

### Common genes between ASD GWAS and developmental brain disorder database (DBD)

**Fig. 2:**
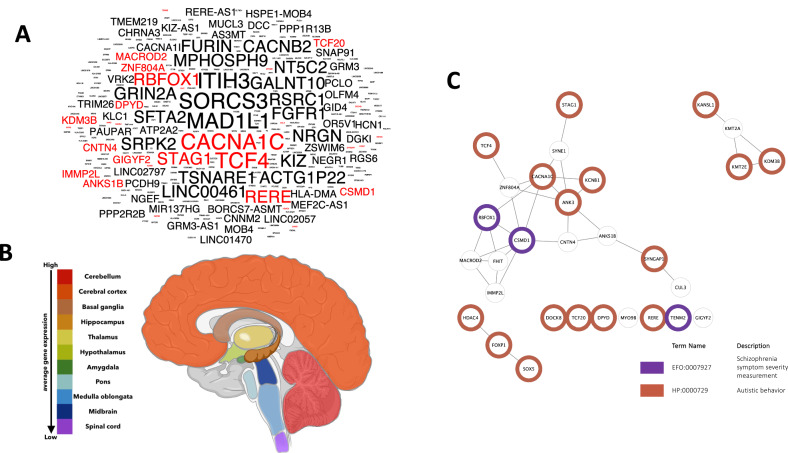
ASD GWAS and DBD genes. **A** The word cloud of GWAS reported genes associated with ASD. Genes shared with DBD are highlighted in red. Same as Fig. 1A, the font size indicates the frequency of gene mentions in publications, with a larger font size indicating a higher number of reported occurrences. The strongly associated genes have been plotted in Supplementary Fig. 2B. **B** RNA expression levels of ASD genes across brain anatomical regions. Overlapping genes of GWAS and DBD were used for creating the gene list (*n* = 30) and the average gene expression level of each brain region was calculated. The “hot” colors represent higher expression levels of the specific brain region and “cold” colors lower expression levels. The graphical image was created with Biorender.com. **C** Gene network of the common gene list of GWAS and DBD. Color-labeled genes are significantly enriched in either category of Autism (*p* = 6.82e-19) or Schizophrenia (*p* = 2.9e-04).

Moreover, we sought to investigate the overlap between our catalog of genes associated with ASD (not the intersection between ASD and SCZ GWAS genes) and those present in the DBD (Fig. 2A and Supplementary Fig. 2B). The DBD includes information obtained through exome and genome sequencing, chromosome microarray analyses, and copy number variation studies. It covers six different developmental brain disorders, including ASD and SCZ, and provides comprehensive phenotypic details that aid in the detection of pathogenic loss of function variants (the recent version includes a total of 7 disorders with the addition of Cerebral Palsy). We identified 30 genes that were shared between ASD GWAS genes and DBD genes. Notably, the top reported ASD GWAS genes CACNA1C, TCF4, STAG1, RBFOX1, and RERE were among this shared gene list. We next used the RNA consensus tissue gene data to determine the brain regions that may be severely impacted by ASD from the human protein atlas (HPA). With the overlapping gene list of genes shared between ASD GWAS and DBD, the average gene expression was computed for each tissue. The findings were represented using color-coded visualization, demonstrating the distinct brain regions influenced by ASD (Fig. 2B). The cerebellum exhibited the highest expression level, while the spinal cord displayed the lowest expression level, with a 37% decrease compared to the cerebellum. Similarly, we also mapped the 78 common genes between ASD GWAS and SCZ GWAS (see Fig. 1A) to the respective brain regions. Contrastingly, here we found cerebellar cortex had the highest expression of these genes followed by basal ganglia, hippocampus, and cerebellum (Supplementary Fig. 3C).

Furthermore, we analyzed which of the genes were associated with autistic vs. schizophrenic behaviors as illustrated in Fig. 2C. Sixteen genes exhibited significant enrichment for “Autistic behavior” (*p* = 9.7e-16). In contrast, a more limited enrichment was found for “Schizophrenia symptom severity measurement” (*p* = 0.0003), with only three genes displaying significant associations (RBFOX1, CSMD1, and TENM2). Thus, it appears that the resulting gene list is more likely to be associated with autistic behavior. In addition, network analysis revealed the presence of three distinct gene clusters. In particular, two smaller clusters, consisting of three and four genes, respectively, showed significant associations with autistic behavior. Conversely, the largest cluster, comprising 16 genes, showed pronounced associations with both ASD and SCZ.

### iPSC models and patient-derived neuronal phenotypes in ASD compared to SCZ

**Fig. 3:**
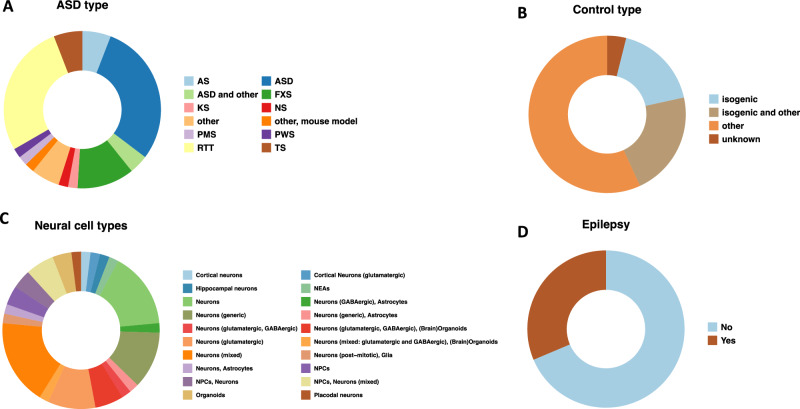
Meta-analysis of iPSC models of ASD. Donut plots (**A**–**D**) of the summary statistics of the collected 51 publications of iPSC models. Four categories (ASD, Control, Neural cell types, and Epilepsy) describe the frequency of specific patients and procedural methods in iPSC models. **A** ASD types with abbreviations of ASD non-syndromic autism spectrum disorder, RTT Rett Syndrome, TS Timothy syndrome, FXS Fragile X syndrome, AS Angelman syndrome, PMS Phelan-McDermid syndrome, KS Kleefstra syndrome, PWS Prader-Willi syndrome, NS Nonsyndromic autism, **B** Control types; **C** Neural cell types with abbreviations of NPCs Neural progenitor cells and NEA Neuroepithelial aggregates, and **D** Publications with patient epilepsy status (yes or no).

We further performed a meta-analysis of previously published work using iPSC models (see methods) (Supplementary Table 1) for ASD, as performed previously for SCZ [7]. Initially, we segmented the data to see the different ASD types (Fig. 3A). The majority of the data is classified as “ASD” (29.4%), followed by Rett Syndrome (RTT, 27.5%), and Fragile X syndrome (FXS, 11.8%). It is important to note that only about 5–10% of ASD cases are known to co-occur with monogenic syndromes such as FXS, Rett syndrome, and Timothy syndrome [63]. In the studies we analyzed, approximately 39.2% of the iPSC models had isogenic lines for controls (Fig. 3B). Also, most of the studies were performed on neurons or neural progenitor cells (NPCs) (Fig. 3C). Some studies were also performed on 3D-brain organoids derived from the iPSC of ASD patients (Fig. 3C). The distribution into neuronal types of the analyzed studies shows that the majority of studies were performed on excitatory (glutamatergic neurons) or mixed neurons with, and only a few studies on GABAergic neurons (Fig. 3C). It is interesting to note that more than 25% of the ASD patients in the studies also had epilepsy (Fig. 3D). The cell type distribution from which the reprogramming was performed is shown in Supplementary Fig. 4A. The vast majority of the studies started by reprogramming fibroblasts (52.9%). The reprogramming was usually performed with integrative methods like retrovirus (50.9%) and lentivirus (35.3%), as well as with integration-free method like Sendai virus (21.6%) or Nucleofection (Supplementary Fig. 4B).

**Fig. 4:**
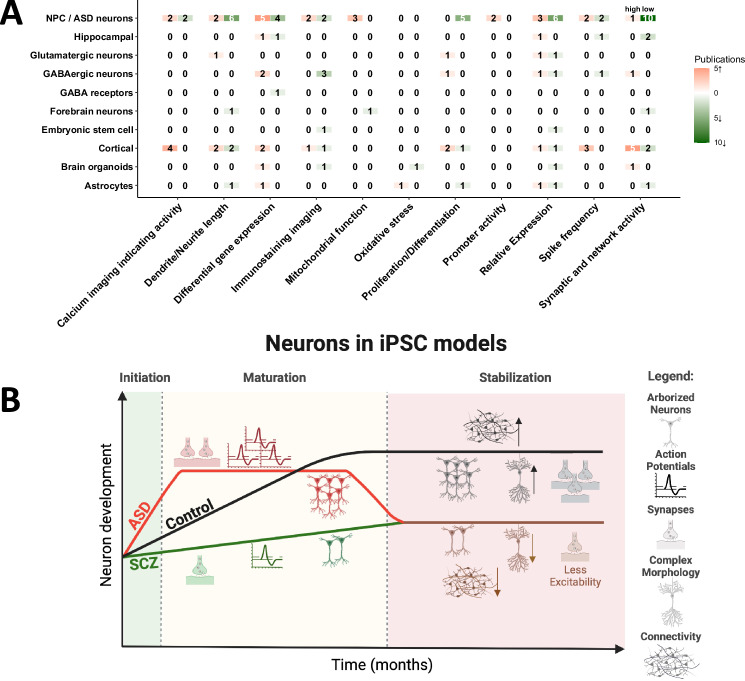
Summary of iPSC studies on ASD and a comparison to SCZ. **A** Heatmap of the number of publications reported activities of specific phenotypes in iPSCs models. If the number of publications is higher, the assigned color becomes darker. With reported high regulation (↑), the color becomes redder, and with low regulation, it becomes green (↓). The color white with the number “0” implies no indication. **B** Schematic of neuron progression over months in reported iPSC models [27, 45–47]. Time was divided into three phases (initiation, maturation, and stabilization). As the neurons develop, the curve also increases. The control curve describes the normal course of neuronal development and serves as a comparison for the ASD and SCZ curves. The arrows (↑↓) next to the figures describe whether this development has increased or decreased. The graphical image was created with Biorender.com.

Figure 4A provides a summary of the phenotypes that were found using the iPSC models. The studies report changes such as differentiation rate, neurites’ length, and differential gene expression. Out of 51 publications, 19.6% reported low synaptic and network activity in neurons derived through NPCs from ASD patients. In addition, 11.8% reported shorter dendrites and neurites and 6% reported increasing associations with mitochondrial function in the neurons derived from the ASD patients. However, the direction is reversed when specifically examining cortical neurons. Five out of seven publications reported increasing synaptic and network activity and likewise, other publications could detect increasing spike frequency and increased activity in calcium imaging [27, 45–47]. Differential gene expression analyses showed a trend toward increased regulation of genes [45, 47].

Figure 4B summarizes the functional phenotypes that are usually reported. The neurons derived from ASD patients usually start with an expedited maturation [27, 46] compared to the control neurons. This includes increased sodium and potassium currents, hyper-excitability, more arborized neurites, and even more synaptic connections initially. However, as the neurons mature, they lose their synaptic connections, have reduced currents, are less excitable, and are less arborized compared to the control neurons derived from individuals without the disorder [27, 47]. Interestingly, the neurons derived from the SCZ patients have a different trajectory but in the later time points, they end up with a similar phenotype [45]. The SCZ neurons start less arborized, are less excitable, have decreased sodium and potassium currents, and have less synaptic activity compared to the control neurons. When the SCZ patient-iPSC-derived neurons develop, they always lag behind the control neurons and when they are mature [45], many of their functional and morphological phenotypes are similar to the neurons derived from the ASD patients (Fig. 4B).

## Discussion

To date, the potential causal common genetic variants of ASD and SCZ have been obscure. Moreover, most previous studies examining the genetic correlations between these two disorders have predominantly focused on populations of European ancestry. Hence, to gain better insights, we performed this study with the extensive analysis of variants reported in the GWAS catalog [34, 35] for patients with ASD, which allowed us to examine diverse ancestries and then compare them to the data from patients with SCZ (also from diverse ancestries). In this way, we were able to find that 74% of the strongly associated ASD genes are also reported in SCZ. Our further analysis of the strongly associated common genes revealed that 45% of the variants are shared between both disorders. Previously, there has been an empirical demonstration of the co-occurrence of ASD and SCZ and hence an overlap of the two in specific population groups [64, 65]. Our analysis, therefore, provides additional support to the assertion that ASD patients may have an increased risk of SCZ [13, 66]. Another important finding from our study is the identification of specific or unique variants in the highly common genes. We discovered that out of 17 highly common genes between ASD and SCZ; seven genes had eight variants that were specific for ASD. Similarly, 16 genes (except NRGN) had multiple variants that were specific for SCZ. Exonic mutations in some of these genes leading to loss of function are known to cause monogenic syndromes. For example, mutations in CACNA1C and TCF4 are known to cause Timothy syndrome [67] and Pitts-Hopkins syndrome respectively [68]. Interestingly, SORCS3, CACNA1C, and CACNB2 (3 of the 17 highly common genes) are also known to be part of the pre- and post-synaptic complexes [69]. A few reports have demonstrated how differences in the variation loci within the same gene (usually in high penetrance rare variants) can lead to changes in the severity of the disorder and even to a different disorder [70–72]. Given the significant role of these highly common genes in regulating essential functional and cellular processes in the nervous system, it is also important to characterize and dissect the cumulative impact of small-effect common gene variants at the cellular level and how these may discern the pathophysiology of ASD and SCZ.

Further, to understand the functional role of GWAS-identified genes, we also searched for the overlap of these genes with the DBD (Developmental Brain disorder) genes. DBD has an exhaustive list of potentially causal genes from neurodevelopmental disorders including Intellectual disability, ASD, ADHD, BD, SCZ, and epilepsy along with phenotypic information. These overlapping critical genes were expressed in different brain regions but a high percentage of them were expressed in the cerebellum, followed by cortex, basal ganglia, hippocampus, and thalamus (top five brain regions). The cerebellum, basal ganglia, and cortex (motor cortex) along with the thalamus are known to coordinate motor-related functions [73]. The hippocampus, cortex, and basal ganglia are important for cognition, memory, and learning as well as language and social communication. Interestingly, while mapping the overlapping genes between ASD GWAS and DBD genes to the brain anatomical regions, we see an overrepresentation of brain areas involved in motor coordination and regulation (Fig. 2B). Though motor impairment is frequent in ASD (affecting approximately 80% of ASD children), the DSM-5 criteria do not include it as a primary characteristic, except for repetitive behaviors [74, 75]. Motor impairments in ASDs include deficits in both fine and gross motor skills, eye movement-related abnormalities, unstable posture, as well as reduced gait, balance, and coordination problems [75, 76]. Several studies have also proposed motor impairments as an early diagnostic feature of ASDs within 1–2 years of age [76–78]. In contrast, when we performed a similar analysis with overlapping ASD & SCZ variants, the representation of the cerebellum was reduced to the fourth place (Supplementary Fig. 3C). SCZ is also characterized by motor abnormalities like catatonia, which involves a range of motor disturbances such as stupor, rigidity, posturing, and bizarre movements [79]. Other motor symptoms may include dyskinesia and psychomotor agitation or retardation. However, in contrast to ASD, motor symptoms in SCZ are often considered secondary to the primary psychotic symptoms [80]. For example, catatonia is only prevalent in approximately 25% of cases [81, 82]. Hence, from this analysis, we can infer that even with a high genetic overlap between ASD and SCZ, the genetic variations can interact and affect different brain areas and circuits resulting in distinct symptoms or behavior in patients.

iPSC-based models offer an alternative to understanding the effect of genomic mutations and associations on neural cells [7, 26, 29, 83–86]. The majority of studies investigated NPCs and neurons derived from ASD patient-specific iPSC in vitro and only a few have studied the role of glial cells from ASD patients using iPSC technology. This dearth of studies on glial cells results from a delay in the availability of good differentiation protocols for microglia, oligodendrocytes, and astrocytes, which are currently an emerging area of study. Glial cells, play a significant role in the central nervous system and are involved in various neuropsychiatric disorders including ASD and SCZ [87]. Astrocytes and microglial cells are crucial for synaptic regulation including synaptogenesis and synaptic pruning [88–90]. In addition, astrocytes and oligodendrocytes play a central role in neuronal migration, differentiation, maturation, and neuronal circuit formation [89, 91, 92]. ASD and SCZ have been shown to involve dysregulation of glial cell activity leading to altered synaptic regulation [93, 94]. Moreover, both ASD and SCZ involve neuroinflammatory processes, where glial cells, and especially astrocytes and microglia, have been reported to play a central role [95, 96]. Chronic neuroinflammation can affect neuronal development, synaptic plasticity, and neurotransmission, contributing to the symptoms of both disorders [96].

The most prominent change that was common across several ASD patient iPSC-derived neuron studies was alteration in synaptic activity. It may seem confounding that some of the studies reported an increase while some reported a decrease in synaptic activity. These differences could in part be due to the different types of neurons studied (cortical, hippocampal, GABAergic, and mixed neuron cultures). Other critical reasons for the variability reported could be the time-point of differentiation studied. Recently, Brant et al. [27] and Hussein et al. [46] have shown how neurons derived from ASD patients start with a higher network connectivity compared to a control network derived from individuals who do not have ASD, but end up with a less connected network when they are maturing. The observation of early hyperexcitability in iPSC-derived neurons of ASD patients is correlated and further supported by a functional magnetic resonance mapping (fMRI) study of 110 children where they found hyperconnectivity in different brain regions in ASD children aged between 7 and 13 years [97]. Hyperconnectivity in the prefrontal cortex at early stages of development has also been demonstrated in a mouse model of cognitive impairment and autism [98].

From the iPSCs-based studies of ASD and SCZ, we see a different trend of phenotypes emerging for these two disorders [7, 31, 42, 43, 46, 99]. The neurons from ASD patients mature earlier with increased arborizations [47] and exhibit electrophysiological properties earlier than controls in earlier time points studied [42–44, 46, 100]. However, gradually they show deteriorating electrophysiological properties at later time points. In the case of SCZ, the neurons are morphologically less arborized as well as exhibit inferior electrophysiological properties throughout the entire differentiation time compared to controls [45] (Fig. 4B). This means that initially the two disorders start with opposite phenotypes, but eventually, the neurons derived from these two disorders exhibit similar phenotypes when they are at a more mature stage. The possible mechanism behind this could be an imbalance in the excitatory-to-inhibitory ratio (E/I). The E/I ratio may be altered owing to changes in the activity of excitatory neurons (glutamatergic neurons) or the activity of inhibitory neurons (GABAergic neurons), or due to the change in the number of excitatory or inhibitory neurons [101]. Additionally, the E/I ratio could also be influenced by the number of glutamatergic or GABAergic synapse formation [102]. An altered E/I ratio has also been demonstrated previously in both ASD and SCZ, and some reports demonstrate an opposite direction of the change in these disorders [103, 104]. Based on this evidence, ASD may be characterized by an early increase in the E/I ratio and later a decrease in the E/I ratio at a more mature stage compared to controls. Similarly, SCZ may be characterized by a decreased E/I ratio compared to the control [105]. Another possibility could be an imbalance in the regulation of synaptogenesis and synaptic pruning. Synaptogenesis results in the formation of new synapses which occurs largely during early development but also continues throughout the lifespan [106]. Synaptic pruning, on the other hand, results in refining the synaptic connections by removing undesirable or excess synapses [106, 107]. Previous evidence in animal models and postmortem brain samples indicates dysregulation of synaptogenesis and synaptic pruning in ASD and SCZ [108, 109]. While ASD has been linked to increased synaptogenesis, SCZ has been shown to have increased synaptic pruning [107, 108]. We speculate that increased synaptogenesis in ASD occurs during early development followed by enhanced synaptic pruning in later stages compared to controls. In SCZ, we presume there is decreased synaptogenesis followed by increased synaptic pruning in comparison to controls. This may be consistent with epidemiological evidence showing that the co-occurrence of SCZ in ASD significantly increases between the ages of 18 to 35 [110], possibly due to enhanced synaptic pruning during this period. As a result, what we observe through iPSC modeling may also serve as a model for the overlap between ASD and SCZ in development.

Our results should be considered in light of some limitations, in light of the difference in the scale of GWAS of ASD and SCZ both in terms of the number of studies (17 ASD studies vs. 86 SCZ studies) and the smaller cohort recruitment in the case of ASD. Additionally, it should be noted that the cerebellum and the cerebral cortex have the largest number of cells [111] and this may influence the distribution of expression that we observed in the variants. Interestingly, however, when focusing on ASD variants, the area in the brain that has the highest expression is the cerebellum, but when focusing on common variants to ASD and SCZ, the cerebral cortex is the most affected area. Furthermore, the majority of the neuronal phenotypes and pathophysiology investigated in both ASD and SCZ have been in iPSC models of rare structural variants, with very few in idiopathic patients. Moreover, our approach to finding the common genes and cellular phenotypes between ASD and SCZ based on the consensus and replication across different studies could have some limitations due to miscellaneous reasons. For instance, the reporting of rare variants in iPSC studies may be biased due to groups that are more well-funded and therefore overrepresented. Future genome-wide studies in ASD with a larger cohort may help to identify additional risk genes. Importantly, more iPSC-based studies in idiopathic ASD and SCZ patients, as well as comparisons with patients with rare variants, are required.

## Conclusion

In conclusion, our study of common genomic variants and the iPSC-derived neuronal pathophysiology of ASD in comparison with SCZ hints at the presence of an initial diverging neuronal pathophysiology that upon maturation converges into a similar pathophysiology despite a significant overlap of genetic associations between the two disorders. This contributes to the prevailing debate about the pleiotropy of genetic factors and the difficulties in identifying causal genetic pathways for neurodevelopmental disorders [23]. Moreover, considering ASD and SCZ as clinically heterogeneous groups, integrating genomic and cellular studies in model systems along with patient stratification could potentially help obtain a deeper understanding of the two disorders.

## Supplementary information

Supplementary Figures 1-4

Supplementary Table 1

## Data Availability

The data that supports the findings of this study are available upon reasonable request from the authors.

## Code availability

The R and MATLAB codes used for statistical analysis and figure generation are available upon reasonable request from the authors.
