# Supplementary material for: Uncovering convergence and divergence between autism and schizophrenia using genomic tools and patients’ neurons
Source: Mol Psychiatry. 2024 Sep 5;30(3):1019–28. doi: 10.1038/s41380-024-02740-0 (PMC11835745; doi:10.1038/s41380-024-02740-0)
Supplement: Supplementary file 1 — Supplementary Figures 1-4 [file 41380_2024_2740_MOESM1_ESM.pdf]

Supplementary figure 1

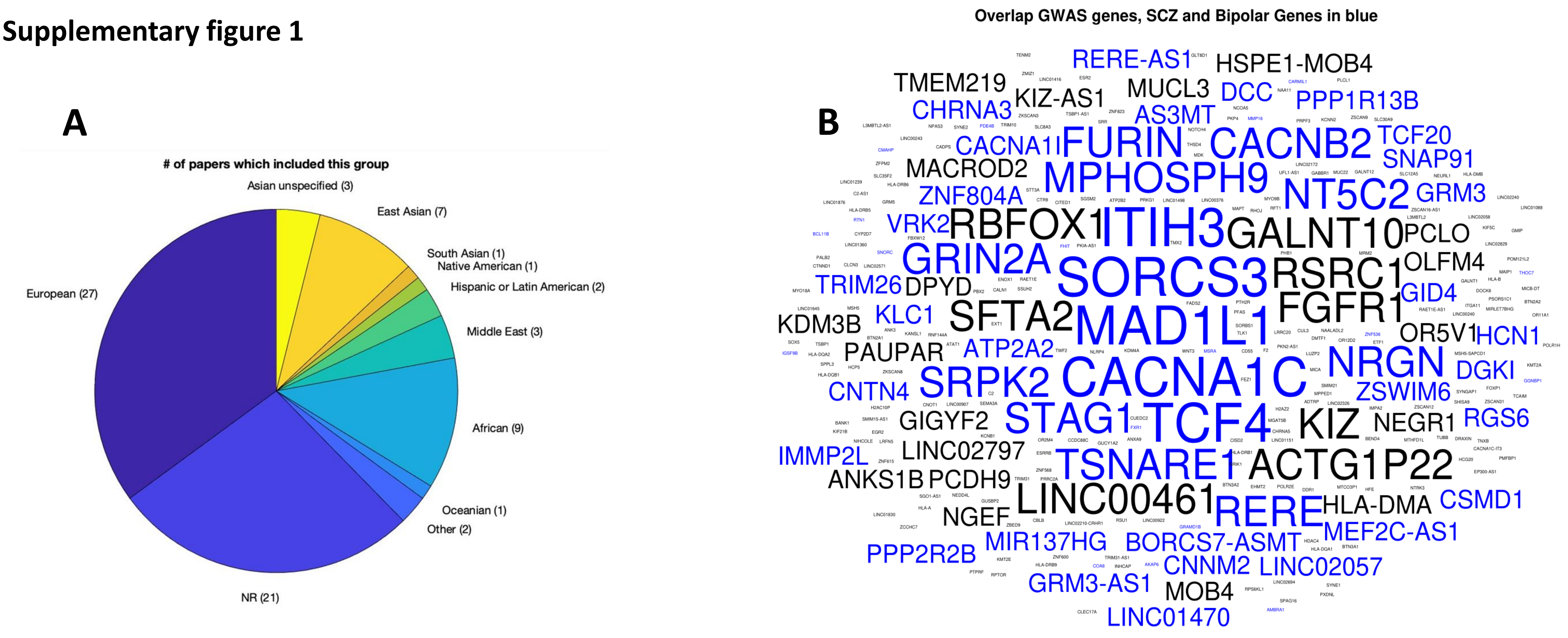

(A) Pie chart distribution of GWAS performed from different ethnic groups across different GWAS publications. The studies table was exported from GWAS with the key word “autism spectrum disorder” (.csv format). Note that some studies reported more than one ancestry and hence more than 17 studies.

(B) The word cloud of GWAS-reported ASD genes common with SCZ and Bipolar disorder. 64 GWAS-reported genes common with SCZ and bipolar disorder are highlighted in blue. The font size indicates the frequency of gene mentions in publications, with a larger font size indicating a higher number of reported occurrences.

Supplementary figure 2

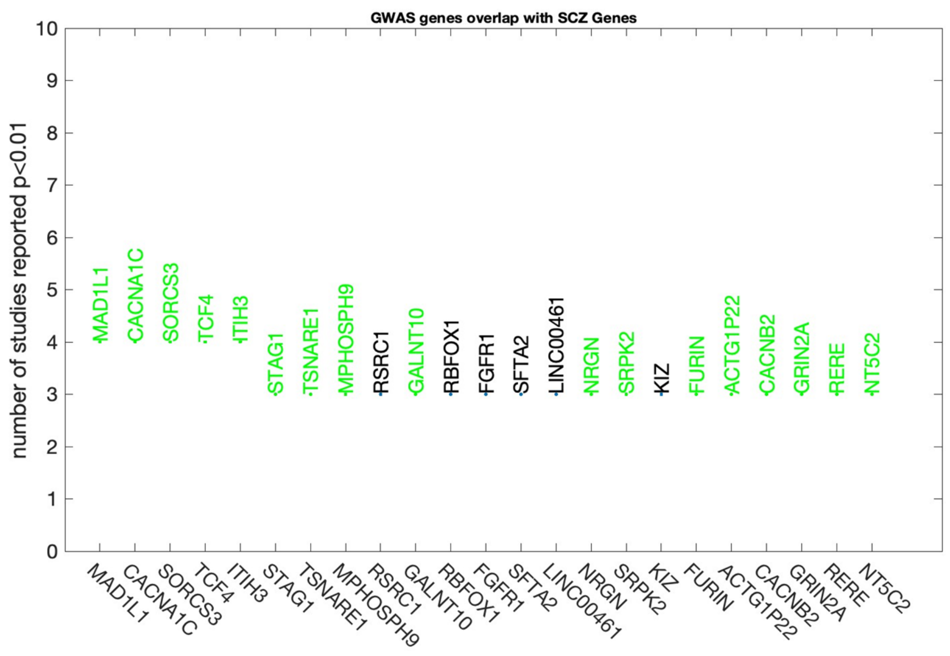

(A) Number of studies reported genes in GWAS associated with ASD (≥3). The 23 genes associated with ASD have been counted to be most occurring in GWAS. The green-labeled genes have been also reported to be associated with SCZ

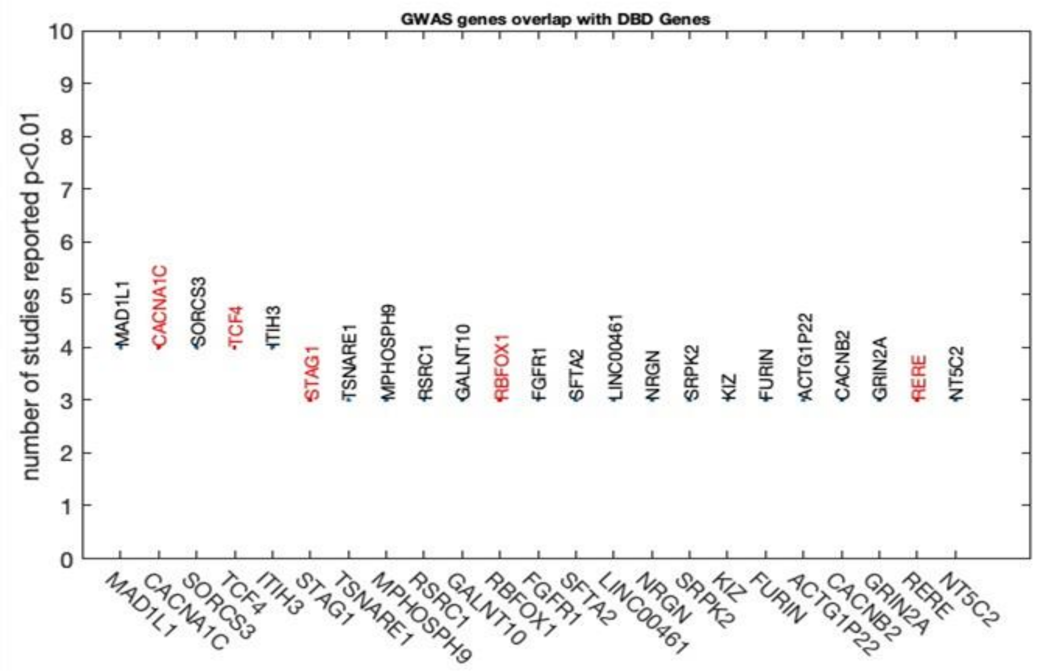

(B) Number of studies reported genes in GWAS associated with ASD (≥3). The red labeled genes been reported in DBD database.

Supplementary figure 3

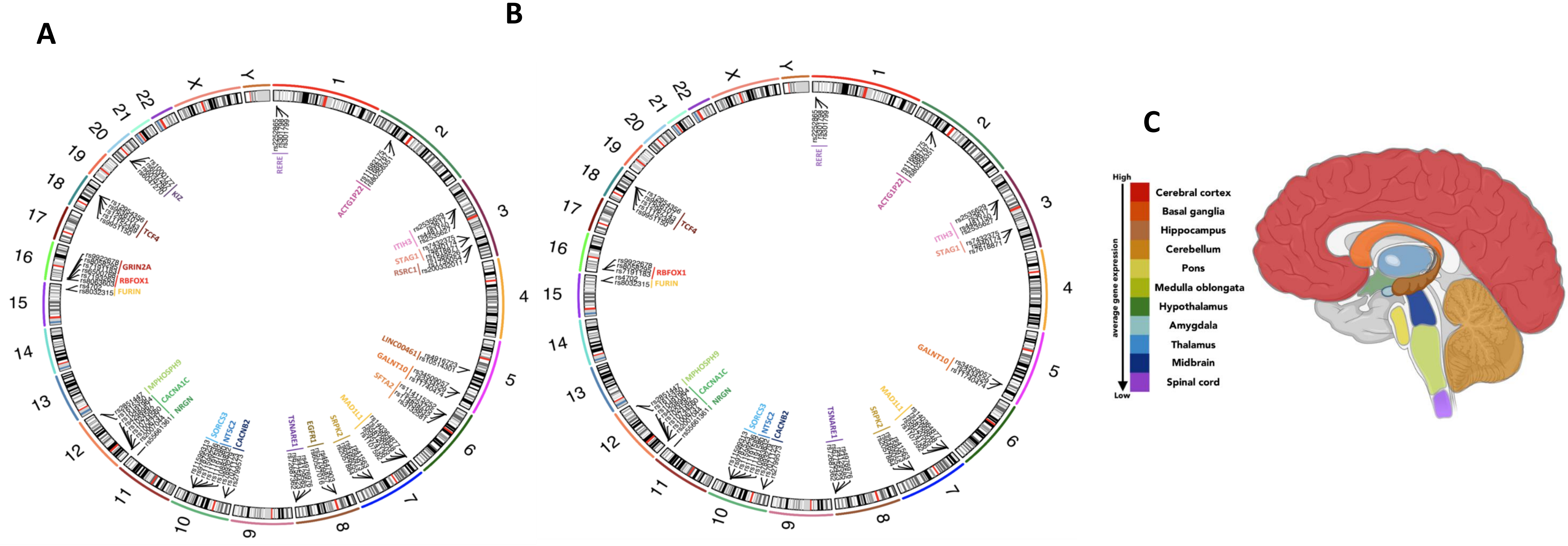

CIRCOS plots of the distribution of SNPs in **(A)** ASD and **(B)** in shared ASD and SCZ genes. Location of each SNP associated with ASD in human chromosomes.
(A) 23 most reported ASD genes with 82 SNPs in GWAS and **(B)** 17 shared strongly associated genes in ASD and SCZ with 63 SNPs in GWAS.

(C) RNA expression levels of ASD vs SCZ genes across brain regions. Overlapping GWAS genes of ASD and SCZ were used for creating the gene list and the average gene expression level of each brain region was calculated. The color bar represents highest and lowest expression of genes across brain regions. The graphical image was created with BrainNet [46] and Biorender.com

Supplementary figure 4

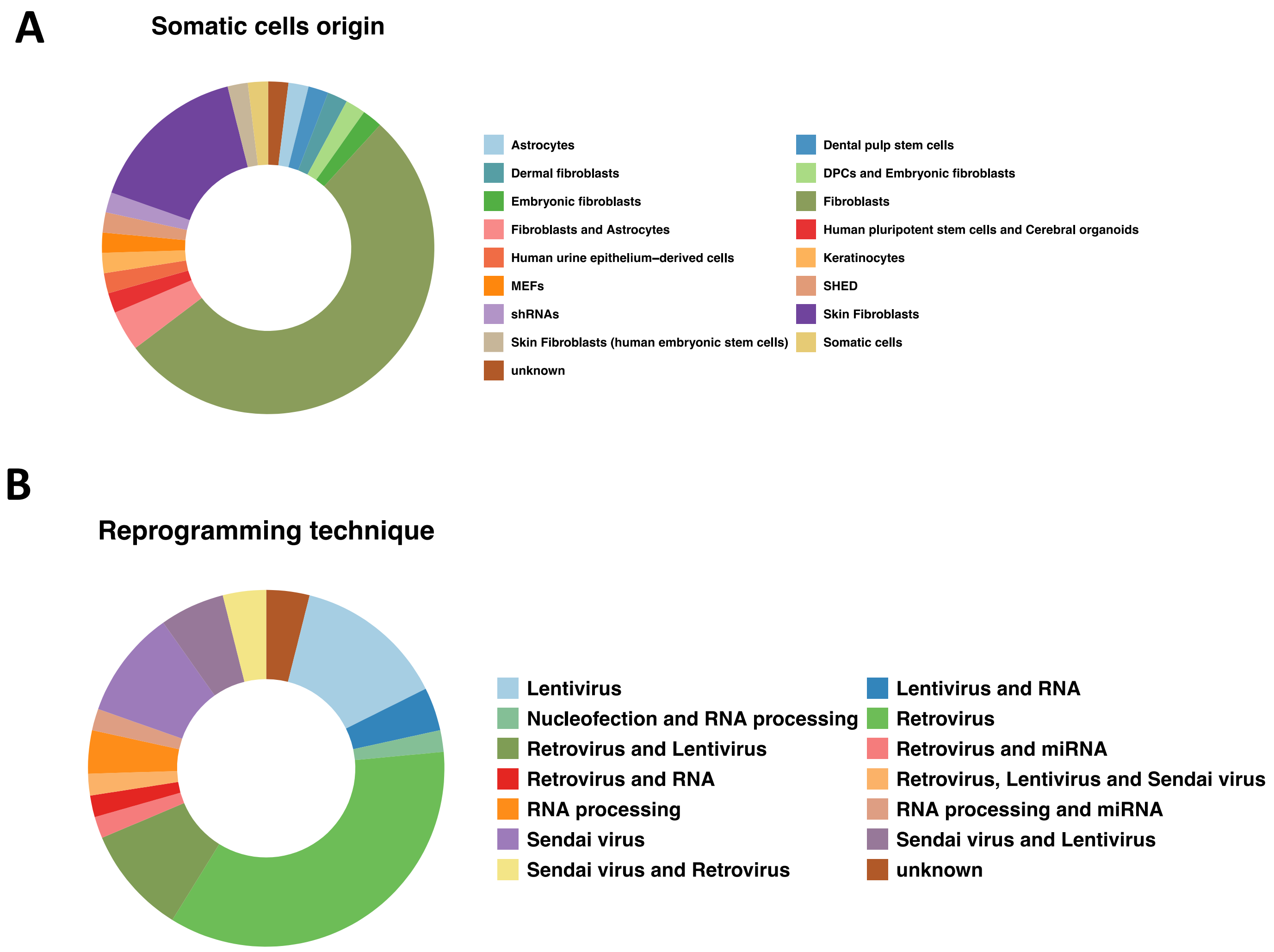

Continued from Figure 3. Donut plots of the summary statistics of the collected 51 publications of iPSC models. (A) Reprogramming technique (B) Somatic cell origin, describe the frequency of specific patients and procedural methods in iPSC models.
